# From genes to genomes in the clinic

**DOI:** 10.1186/s13073-015-0200-0

**Published:** 2015-07-29

**Authors:** Joris A. Veltman, James R. Lupski

**Affiliations:** Department of Human Genetics, Donders Centre for Neuroscience, Radboud University Medical Centre, Geert Grooteplein 10, 6525 GA Nijmegen, The Netherlands; Department of Clinical Genetics, Maastricht University Medical Centre, Universiteitssingel 50, 6229 ER Maastricht, The Netherlands; Department of Molecular and Human Genetics, Department of Pediatrics, and Human Genome Sequencing Center, Baylor College of Medicine, One Baylor Plaza Room 604B, Houston, TX 77030 USA; Texas Children’s Hospital, Houston, TX 77030 USA

## Abstract

Next-generation sequencing is revolutionizing medical genetics and in the near future will pervade all medical fields. To maximize the potential clinical utility of this approach, global data sharing and phenotyping are needed, and the role of the geneticist in the interpretation of variation is vital.

## Towards single-test genomics

Medical genetics is experiencing an exciting and disruptive technological revolution. Next-generation sequencing (NGS) technologies are enabling simultaneous sequencing of all relevant disease genes, the exome or even the entire genome of patients seeking a molecular diagnosis. This can be performed on small sample volumes and even single cells with near-perfect accuracy, in days rather than months, and at more and more affordable pricing, allowing widespread implementation in the clinic [1]. This is an important moment in the history of medical genetics, because for the first time we can study a person’s entire genome in our quest to find the genetic causes underlying disease and/or those potentially having an impact on clinical response to treatment.

Genome sequencing, although still imperfect, will enable the detection of all types of genomic variation in a single experiment, including point mutations or single nucleotide variants, insertion-deletions, repeat expansions and larger structural variations such as copy number variants and copy-number-neutral inversions and translocations. Although genome sequencing is currently more expensive than targeted disease gene or exome sequencing as calculated on a per test basis, this may not be the case when calculated on a per patient basis. Pilot studies have already demonstrated that genome sequencing can reveal causative mutations or structural variations missed by other genetic tests, including Sanger sequencing, exome sequencing and genomic microarrays [2, 3]. Regardless of which NGS approach is used, it is evident that variant identification is becoming more and more readily attainable, whereas variant interpretation in each individual patient remains a major challenge [4, 5]. This has considerable consequences for medical genetics laboratories, where there will be less need for expertise in laboratory data generation and increased demand and necessity for expert data analysis and interpretation.

Single-test or comprehensive genetic testing, such as that provided by genome sequencing, has many important advantages in the clinic. It allows automated laboratory procedures and standardization of variant calling and reporting. No longer is a subjective clinical decision required to determine which genetic test to perform and in which patient. Instead, implementation of a single genetic test is sufficient. It is conceivable that best practice will evolve to a point at which genome sequencing will be performed before a patient even visits a clinician. This may enable the clinician to capitalize on the genomic information immediately and use it to form a diagnosis.

Comprehensive genomic sequencing requires a different skill set for clinical geneticists and other medical professionals using genetic data. The genomic data from patients, collected in a standardized format all over the globe, will be enormously valuable to improve our understanding of variant pathogenicity and disease susceptibility. Data sharing on a global scale is therefore essential to improved understanding of genotype–phenotype correlations [6]. Global data sharing is not just a lofty goal but rather a requirement for understanding human biology and deviations from homeostasis or health; it transcends geographic, political and religious boundaries that can sometimes divide the human race. These genomic data are, however, useful only if combined with phenotypic information collected in a standardized manner as well. It is remarkable to think that genome sequencing itself may soon turn out to be the easy part of the equation. Standardized phenotyping all over the world is likely to be a major challenge required for maximizing the medical knowledge acquisition from personal genomic variant information. This requires clinicians to adapt their clinical practice and potentially adopt and incorporate novel phenotyping tools.

Clearly, there are ethical and legal issues related to medical genome sequencing. These are mostly related to ownership and privacy of personal data and the risk of detecting clinically relevant genomic variants unrelated to the original clinical conundrum and concern for which the patient visited the clinician [7]. Patients need to be protected from misuse of their genomic data and need to be adequately counseled so that they can understand the information contained in their genome and the current limitations of medical knowledge. In this regard, an obligation of the community of geneticists and genomicists will be to educate the physicians, patients and public about DNA, the genetic code, genomics and its role in human biology, health and disease susceptibility. The growth of genome sequencing capacity is impressive by any measure, with commercial platforms now being able to sequence 10,000–50,000 genomes per year. This has spurred a range of national programs, such as the 100,000 Genomes Project in the United Kingdom (http://genomicsengland.co.uk), FarGen (http://www.fargen.fo/en) in the Faroe Islands, and the precision medicine initiatives in the USA (http://www.nih.gov/precisionmedicine/) and Saudi Arabia (http://shgp.kacst.edu.sa/site/). At the same time, individual medical genetics laboratories around the globe are learning to interpret individual variation obtained from targeted disease gene and exome sequencing in their clinics on a daily basis.

## Clinical genomics

Rapid, accurate, comprehensive and affordable NGS approaches are desperately needed to realize the many promises of personalized or precision medicine. Traditionally, genetics has had a major role in diagnosing rare disease and hereditary forms of cancer, as well as in prenatal testing for Down syndrome. As a result, NGS approaches are first replacing Sanger sequencing and genomic microarrays for these applications because of their higher diagnostic yield and non-invasive application. The higher diagnostic yield of NGS approaches also results in an expansion of medical conditions for which genetic testing is being considered. For many of these disorders, such as immune deficiencies and autism spectrum disorders, it was known that genetics has a role in a significant percentage of cases. Their genetic heterogeneity, however, requires the comprehensiveness of NGS.

A rapid turn-around time is even more important for determining the right cancer therapy, for residual disease monitoring, and in neonatal intensive care units [8]. Sequencing of cancer genomes may soon become a standard diagnostic procedure, similar to a histopathological evaluation, but different in that it can be applied on multiple occasions to determine remission and resistance to therapy. Cancer researchers are accumulating evidence that this will guide individual therapy to the most effective cancer drugs, and speed up drug repurposing as well as novel drug target discovery [9]. Getting the right amount of the right drug to the right patient is a challenge in most of medicine. Pharmacogenetics is currently underused in medicine, even though there are more than 100 drugs for which genetic testing is recommended. The possibility of offering a comprehensive, affordable and rapid NGS-based test is likely to be of great benefit for pharmacogenetics as well [10]. In a prenatal setting, non-invasive screening for trisomies using NGS approaches is only a first step. Pilot studies, although complex and expensive, have shown the possibilities to sequence the genome of the fetus with all its variation [11]. An alternative could be to offer preconception screening to couples, and again NGS approaches are now allowing this to be done comprehensively for all known recessive conditions [12]. Interpretation of these genomic data to predict disease is much more complex in a prenatal or preconception setting than it is in a postnatal setting. Predicting the age of onset or severity of disease is even more complex in a prenatal setting. In addition, for many severe early-onset disorders such as intellectual disability, we have recently learned that the condition is often caused by de novo mutations [3], which would be missed by preconception screening. It is unknown to what extent prenatal or preconception genetic screening will be implemented or sought, but the technology is making these into realistic options.

In conclusion, it is not hard to imagine a near future in which genomics is pervasive throughout medicine, in many subspecialties and in general practice. The human genetics and genomics field will change, but geneticists are needed more now than ever, working together with other medical specialties to provide clinically relevant genomic information and medical knowledge, with a common goal: to improve patient care.
